# Prospecting and informed dispersal: Understanding and predicting their joint eco‐evolutionary dynamics

**DOI:** 10.1002/ece3.8215

**Published:** 2021-10-18

**Authors:** Aurore Ponchon, Alice Scarpa, Greta Bocedi, Stephen C. F. Palmer, Justin M. J. Travis

**Affiliations:** ^1^ School of Biological Sciences University of Aberdeen Aberdeen UK

**Keywords:** breeding failure, breeding habitat selection, conspecific breeding success, environmental changes, population dynamics, social information

## Abstract

The ability of individuals to leave a current breeding area and select a future one is important, because such decisions can have multiple consequences for individual fitness, but also for metapopulation dynamics, structure, and long‐term persistence through non‐random dispersal patterns. In the wild, many colonial and territorial animal species display informed dispersal strategies, where individuals use information, such as conspecific breeding success gathered during prospecting, to decide whether and where to disperse. Understanding informed dispersal strategies is essential for relating individual behavior to subsequent movements and then determining how emigration and settlement decisions affect individual fitness and demography. Although numerous theoretical studies have explored the eco‐evolutionary dynamics of dispersal, very few have integrated prospecting and public information use in both emigration and settlement phases. Here, we develop an individual‐based model that fills this gap and use it to explore the eco‐evolutionary dynamics of informed dispersal. In a first experiment, in which only prospecting evolves, we demonstrate that selection always favors informed dispersal based on a low number of prospected patches relative to random dispersal or fully informed dispersal, except when individuals fail to discriminate better patches from worse ones. In a second experiment, which allows the concomitant evolution of both emigration probability and prospecting, we show the same prospecting strategy evolving. However, a plastic emigration strategy evolves, where individuals that breed successfully are always philopatric, while failed breeders are more likely to emigrate, especially when conspecific breeding success is low. Embedding information use and prospecting behavior in eco‐evolutionary models will provide new fundamental understanding of informed dispersal and its consequences for spatial population dynamics.

## Graphical abstract

Information use affects the evolution of emigration probability and the number of prospected patches. It has to be considered when addressing dispersal as a response of individuals and populations to environmental changes.

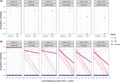

## 1 INTRODUCTION

Dispersal defines the movement of an individual from its natal or current breeding patch to a new one (Clobert et al., 2001). It represents a crucial process in ecology and evolution, since it has a major influence on population dynamics, structure and persistence (Bowler & Benton, 2005; Clobert et al., 2012), gene flow between populations (Ronce, 2007), and species’ range dynamics (Kokko & Lopéz‐Sepulcre, 2006; Travis et al., 2013). Dispersal can be decomposed into three main stages: emigration from the natal or current breeding patch, transience (movement between the two patches), and settlement in a new breeding patch (Clobert et al., 2009). Furthermore, each phase may incur different costs (Bonte et al., 2012; Travis et al., 2012) and involve numerous context‐dependent decisions made at the individual level which drive individual movements (Clobert et al., 2009).

**FIGURE 1:**
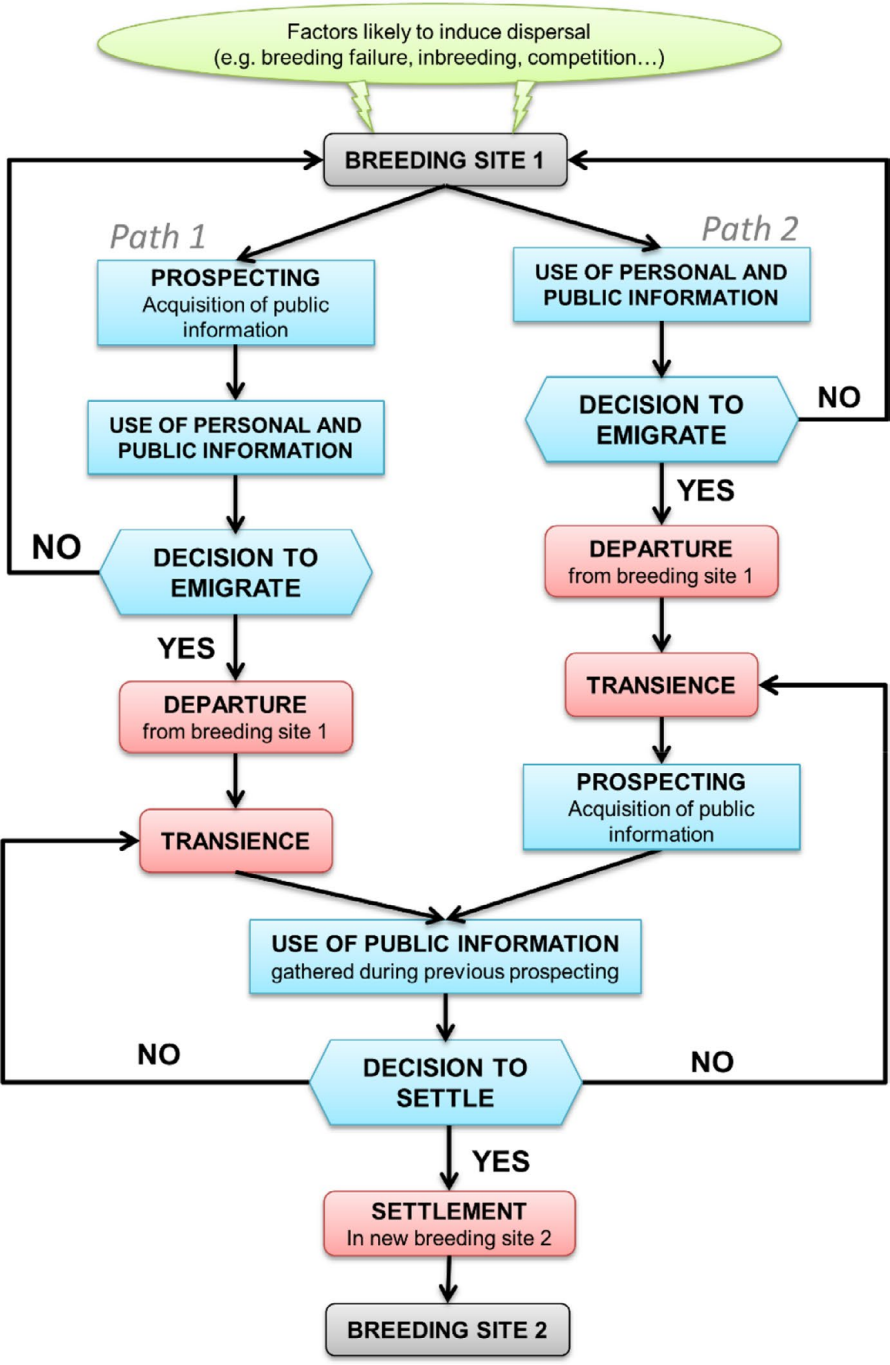
Flowchart of informed dispersal representing dispersal (red) and information acquisition and use in breeding habitat selection (blue). Breeding site 1 can be the natal or current breeding site. Depending on species life cycle, prospecting can occur before (Path 1 ‐ Best‐of‐n strategy) or after emigration decision (Path 2; sequential sampling)

In a variable but predictable environment, many organisms are able to gather and use information to decrease the uncertainty about the quality of their environment and make better decisions (Dall et al., 2005). When they do so in the context of dispersal, they adopt a strategy termed ‘informed dispersal’ (Clobert et al., 2009; Reed et al., 1999). The sources of information on the local environmental conditions are diverse and can be divided into three main types: (1) personal information obtained from the direct interaction of individuals with their environment (i.e., visual, hearing, and chemical cues) and their past experience (i.e., individual breeding success, familiarity with the environment; Dall et al., 2005), (2) social information obtained from the presence or density of conspecifics and defined as conspecific attraction (Stamps, 1988), and (3) social information obtained from the performance of con‐ or hetero‐specifics (i.e., breeding success, quantity and quality of the offspring) and defined as public information (Dall et al., 2005; Danchin et al., 2004; Seppänen et al., 2007). The acquisition and use of information can occur before emigration, when individuals choose whether to leave their current breeding patch, during transience, and/or just before choosing where to settle (Figure 1). Information acquisition often involves prospecting phases, which are visits of individuals to breeding areas or sites where they do not currently breed and where they can gather personal and social information on the local environmental quality to make emigration and/or settlement decisions (Reed et al., 1999). As a result, individuals do not disperse randomly in the landscape, and information acquisition and use are therefore essential to consider for better understanding dispersal processes (Bocedi et al., 2012; Delgado et al., 2011, 2014; Enfjäll & Leimar, 2009; Fronhofer et al., 2017; Ponchon, Garnier, et al., 2015; Schmidt et al., 2015; Vuilleumier & Perrin, 2006). Direct observations in the field, along with the recent development of miniaturized tracking devices, have revealed that dispersal in natural populations can be driven by complex emigration and settlement decision‐making processes (Clobert et al., 2009; Reed et al., 1999). Nevertheless, because they do not involve the same spatial and temporal scales, emigration and settlement phases have generally been studied separately, both in the field and in theoretical models (Clobert et al., 2009).

Regarding the emigration phase, many empirical studies have demonstrated that individuals use both personal and public information to make informed emigration decisions, leading to higher emigration probabilities when individuals fail to breed among failed conspecifics (Boulinier et al., 2008; Danchin et al., 1998; Dugger et al., 2010; Pakanen et al., 2011; Rioux et al., 2011; Robert et al., 2014). Despite the growing empirical evidence of the use of these two types of information, theoretical models have been largely restricted to investigating the evolution of density‐dependent emigration probabilities, when individuals cue only on local conspecific density (Bocedi et al., 2012; Enfjäll & Leimar, 2009; Fronhofer et al., 2017). Those studies have shown that the evolved emigration rate decreased when individuals used patch densities as social information under conditions of high dispersal mortality cost and temporally uncorrelated (i.e., unpredictable) environmental fluctuation (Enfjäll & Leimar, 2009), whereas emigration rates increased when individuals used information in temporally auto‐correlated (i.e., predictable) environments (Bocedi et al., 2012). Bocedi et al. (2012) further showed that the acquisition of costly information on local conspecific density always evolved, especially in predictable environments. Nevertheless, selection rarely favored investment in the acquisition of high‐precision information.

Regarding the settlement phase, empirical studies have shown that many colonial and territorial species including birds (Reed et al., 1999), mammals (Mares et al., 2014; Mayer et al., 2017; Selonen & Hanski, 2010), reptiles (Cote & Clobert, 2007), amphibians (James et al., 2015; Pizzatto et al., 2016), and arthropods (De Meester & Bonte, 2010; Seeley & Buhrman, 2001; Stroeymeyt et al., 2017) gathered public information during prospecting in order to settle in more productive breeding areas. Importantly, the use of public information was often favored over conspecific density and personal information in both emigration and settlement decisions (Aparicio et al., 2007; Betts et al., 2008; Doligez et al., 2002; Forero et al., 1999; Pärt et al., 2011; Ponchon, Chambert, et al., 2015; Ponchon, Iliszko, et al., 2017), outlining its crucial role in informed dispersal.

A few theoretical studies have started implementing dispersal as a complex behavioral process, integrating the different phases of dispersal. For example, Travis et al. (2012) proposed an eco‐evolutionary framework for modeling dispersal, emphasizing the need to model explicitly the three dispersal phases and the associated costs. However, they only briefly mentioned information use and they did not explicitly implement it in their model example. To our knowledge, only one theoretical study explicitly considered the eco‐evolutionary dynamics of prospecting in the settlement phase (Delgado et al., 2014). It highlighted an important interplay between prospecting and dispersal strategies and showed that selection for informed dispersal in many cases resulted in lower population abundances and patch occupancies than under random dispersal. However, public information based on conspecific breeding performance was not incorporated, and the authors called for further studies integrating information use in emigration decisions as well. Additionally, two ecological models incorporated information use in both emigration and settlement decisions with a prospecting phase (Ponchon, Garnier, et al., 2015; Schmidt, 2017). They showed that in a changing environment, informed dispersal based on prospecting and public information use led to greater population size and persistence compared to random dispersal or philopatry. However, neither model incorporated the evolution of those informed dispersal strategies.

Overall, there has been a substantial discrepancy between the complex dispersal processes that have been described in natural populations and the ones implemented in evolutionary models. As a result, we currently lack a general understanding of the consequences of informed dispersal for individual fitness and population functioning. To fill this gap, we developed an eco‐evolutionary model that links emigration, prospecting, public information use, settlement, and demography in temporally auto‐correlated environments. We first determined how the number of prospected patches evolved depending on the use of personal and public information, the patch selection strategy, and the cost of prospecting. Then, we determined how emigration probabilities and prospecting evolved concurrently.

## 2 THE MODEL

We developed a stochastic individual‐based model that tracks ecological dynamics of populations and evolutionary changes in individual dispersal strategies. The life cycle of individuals is inspired by a long‐lived colonial species, but can be adapted to any species whose populations are spatially structured in a heterogeneous and temporally variable environment.

We constructed a female‐only model with three distinct life stages (juveniles, pre‐breeders, and adults), overlapping generations, and negative density dependence in fecundity. Importantly, we integrated the use of personal and public information in emigration decisions and an independent prospecting phase during which individuals gather public information to make settlement decisions. The dispersal genotype and social environment of individuals can both affect their behavior, of which population dynamics is an emergent outcome. Here, personal information corresponds to individual breeding success (success or failure of an individual at producing offspring), while public information corresponds to local conspecific reproductive success in a given breeding patch at the time of prospecting. All the defined parameters can be found in Table 1, and the source code and output files are freely available at https://github.com/auponchon/Informed‐dispersal‐IBM and are deposited on Zenodo (DOI: 10.5281/zenodo.5534084).

**TABLE 1:** Common values used to parameterize the model in the two examples. Parameters specific to each model example are given in Section 3

Parameters	Abbreviation	Value
Carrying capacity in a patch	*K*_0_	100
Maximum mean number of offspring produced by female	Off_max_	2
Juvenile survival	*S*_J_	0.6
Pre‐breeder survival	*S*_I_	0.7
Adult survival	*S*_A_	0.85
Standard deviation for the environment	*σ*	1
Temporal autocorrelation coefficient	*α*	0.8
Mean age at recruitment	*R*	5
Mutation rate	*μ*	0.01
Mortality cost of prospecting per patch	*M*	0.01

### 2.1 The environment

Previous theoretical models have demonstrated that informed dispersal can only evolve when the environment is variable but predictable in time, that is, temporally auto‐correlated (Boulinier & Danchin, 1997; Doligez et al., 2003). Therefore, we assume a spatially structured population existing within 25 discrete breeding habitat patches, each of which has an independent local and temporally auto‐correlated environmental quality *Q_x_*_,_ *_y_*_,_ *_t_* that varies annually. At time *t* = 0, each patch *x*,*y* is given a local environmental quality *Q_x_*_,_ *_y_*_,0_ based on a value *w_x_*_,_ *_y_*_,0_ drawn from a normal distribution *N*~(0,*σ*) so that:

(1) $$Q_{x,y,0}=w_{x,y,0}$$

At *t* + 1, the local environmental quality $Q_{x,y,t+1}$ depends on $Q_{x,y,t}$ and *w_x_*_,_ *_y_*_,_ *_t_* is resampled from a normal distribution *N*~(0,*σ*) and associated with an autocorrelation coefficient *α* so that:

(2) $$Q_{x,y,t+1}=\alpha \times Q_{x,y,t}+w_{x,y,t}\times \sqrt{1‐\alpha^{2}}$$

The carrying capacity of each patch *K_x_*_,_ *_y_*_,_ *_t_*, which is always ≥ 0, is directly affected by the local environment quality so that:

(3) $$K_{x,y,t}=K_{0}+K_{0}\times Q_{x,y,t}$$

Note that changing *σ* changes the amplitude of the fluctuations of the environmental quality (Bocedi et al., 2014). Therefore, we ran preliminary simulations to check how *σ* and temporal autocorrelation *α* could affect the eco‐evolutionary dynamics of populations. As expected, when the temporal autocorrelation *α* was low, random dispersal was dominant and strongly affected the spatial distribution of individuals with weaker density‐dependent effects on fecundity (see Figure S1). Likewise, increasing *σ* increased the range of the environmental quality but this did not affect the patterns observed in the evolution of prospecting. Based on those simulations, we chose a high autocorrelation coefficient (*α* = .8) and a moderate standard deviation (*σ* = 1) to run the different experiments (Table 1).

### 2.2 Reproduction

The annual cycle of individuals in patch *x*,*y* starts with reproduction. Each female *i* produces a number of offspring sampled from a Poisson distribution with a mean *μ*_Off_ given by:

(4) $$\mu_{\text{Off}}=\frac{{\text{Off}}_{\max}}{\left(1+\left({\text{Off}}_{\max}‐1\right)\times \frac{N_{x,y,t}}{K_{x,y,t}}\right)}$$

where Off_max_ is the maximum mean number of offspring produced per female and *N_x_*_,_ *_y_*_,_ *_t_* is the number of adults present in patch *x*,*y* in year *t*. If individuals successfully produce one or more offspring, they are successful breeders. Otherwise, they are failed breeders. The local conspecific breeding success LBS*_x_*_,_ *_y_*_,_ *_t_* is calculated as the ratio of the number of successful breeders to the number of adults *N_x_*_,_ *_y_*_,_ *_t_*_,_ as usually calculated in the field (e.g., Ponchon et al., 2014).

### 2.3 Life stages

The offspring produced are juveniles and have a probability *S*_J_ of surviving to become pre‐breeders the following year (Table 1). The mean age at recruitment, that is, age at which pre‐breeders become adults and attempt to breed for the first time, is generated at birth from a Poisson distribution with a mean *R*. As long as pre‐breeders do not reach age of recruitment, they experience an annual survival probability *S*_I_ (Table 1) and remain pre‐breeders. They neither breed nor disperse. When they recruit and become adult, they can disperse to select their first breeding patch. Adults have an annual survival probability *S*_A_ (Table 1).

### 2.4 Emigration, prospecting, and settlement

Dispersal is modeled in three phases: emigration decision, prospecting, and settlement decision (Figure 1; Path 1). We considered three different emigration strategies: (i) the emigration decision is non‐informed (i.e., all individuals have the same emigration probability *E*); (ii) emigration probability depends on the individual's breeding status: if successful, *E*_succ_; if failed, *E*_fail_, which implies the use of personal information; (iii) emigration probability is a function of both personal and public information (i.e., local conspecific breeding success *LBS_x_*_,_ *_y_*_,_ *_t_*) defined by the following:

(5a) $$E_{\text{fail}\;}=\beta_{\text{fail}}+\alpha_{\text{fail}}\times {\text{LBS}}_{x,y,t}$$

(5b) $$E_{\text{succ}\;}=\beta_{\text{succ}}+\alpha_{\text{succ}}\times {\text{LBS}}_{x,y,t}$$

Parameters defining each emigration strategy (i.e., *E*; *E*_succ_ and *E*_fail_; or *α*_fail_, *α*_succ_, *β*_fail_, and *β*_succ_) can either be fixed for the population or evolving traits depending on the simulation scenario (see Section 4).

When pre‐breeders become adult, they can choose where to recruit and breed for the first time. As they have not reproduced yet, they have no past breeding performance. Nevertheless, they are assigned the same emigration probability as failed breeders, *E*_fail_, as they are assumed to use conspecific breeding success to make their emigration decision.

If individuals decide to emigrate, they prospect by randomly selecting a set of patches, corresponding to the number of prospected patches determined by an additional evolving trait, *N*_p_. The prospected patches are ranked according to their breeding success and assigned a probability of being chosen derived from their current breeding success. The probability *p_i_* to select the *i*th ranked prospected patch is determined according to three alternative patch selection processes reflecting the differential ability of individuals to discriminate patches with the highest breeding success: (i) an inaccurate process, where $p_{i}={\text{LBS}}_{i}/\sum_{i}\text{LBS}$, so that individuals are somewhat likely to choose the best prospected patches, (ii) an accurate process, where $p_{i}=e^{50*{\text{LBS}}_{i}}/\sum_{i}e^{50*{\text{LBS}}_{i}}$ so that individuals will tend strongly to settle in the best prospected patch but could still choose other patches with a small probability weighed by local breeding success, and (iii) a deterministic process, where *p*_1_ = 1 and *p_i_* = 0 for all other prospected patches so that individuals always settle in the prospected patch with the highest breeding success (Figure S2). Individuals that do not prospect (*N*_p_ = 0) choose a breeding patch at random. Likewise, as the initial selection of prospected patches is random, a prospecting strategy based on only one patch (*N*_p_ = 1) is equivalent to random settlement. Once individuals have decided to emigrate, they cannot return to their immediately previous patch and must settle in another occupied patch.

### 2.5 Evolution of dispersal traits

Individuals are haploid and carry one locus for each evolving trait determining their emigration probability and their prospecting propensity, depending on the simulated scenario. *E* (random emigration probability), *E*_succ_ and *E*_fail_ (emigration probabilities based only on personal information), and *α*_fail_, *α*_succ_, *β*_fail_, *β*_succ_ (parameters defining the relationship between emigration probability and local breeding success, Equation 5) are all coded by continuous alleles. *N*_p_, the trait defining the number of prospecting patches, is coded by discrete alleles. Offspring inherit alleles from their mother. In scenarios where more than one trait is evolving, loci are unlinked and each subject to a mutation probability *μ* = 0.01 per generation. When a mutation occurs for one of the continuous traits, the allelic value is altered by an increment drawn from a normal distribution *N ~* (0, 0.1), while the allele coding for prospecting propensity is changed by ±1.

Simulations are initialized by assigning each individual a random allele for the number of prospected patches, *N*_p_, from 0 to 24. When one or more continuous traits determining emigration are evolving, each locus is initialized with a random value between 0 and 1, except *α*_fail_ and *α*_succ_ which are initialized with a random value between −1 and 0, assuming a negative relationship between emigration probability and local breeding success (Equation 5).

## 3 MODEL EXPERIMENTS

### 3.1 Evolution of prospecting only

First, we conduct one experiment comprising a set of simulations to determine how different non‐evolving emigration strategies and patch selection processes affect the evolution of prospecting and population spatial structure. Only one trait is evolving, that is *N*_p_. We consider the three different emigration strategies described above: constant emigration probability (*E* = 0.5), emigration depending only on personal information (*E*_succ_ = 0.05 and *E*_fail_ = 0.5), and emigration depending on both personal and public information (*β*_succ_ = 0.05, *α*_succ_ = 0.0, *β*_fail_ = 0.5, *α*_fail_ = −0.45). For each emigration strategy, we implement, in turn, each of the three different patch selection processes: inaccurate, accurate, and deterministic. Finally, for each scenario, we implement either no dispersal mortality cost or a mortality cost *M*_cost_ proportional to the number of prospected patches, namely *M*_cost_ = *M* * *N*_p_, where *M* = 0.01. We ran each scenario over 20,000 years and repeated it 10 times.

### 3.2 Evolution of both prospecting and emigration probability

In a second experiment, we test how the emigration probability and the number of prospected patches concomitantly evolve depending upon the patch selection process and prospecting cost. We conduct these experiments for each of the three emigration strategies that involve different levels of information use. Prospecting evolution and costs are based on the same rules as the previous model example.

Depending on the emigration strategy, one (*E*), two (*E*_succ_, *E*_fail_), or four (*α*_fail_, *α*_succ_, *β*_fail_, *β*_succ_) emigration parameters can evolve independently along with prospecting *N*_p_. We ran each scenario over 20,000 years and replicated it 10 times.

## 4 RESULTS AND DISCUSSION

### 4.1 Evolution of prospecting only

**FIGURE 2:**
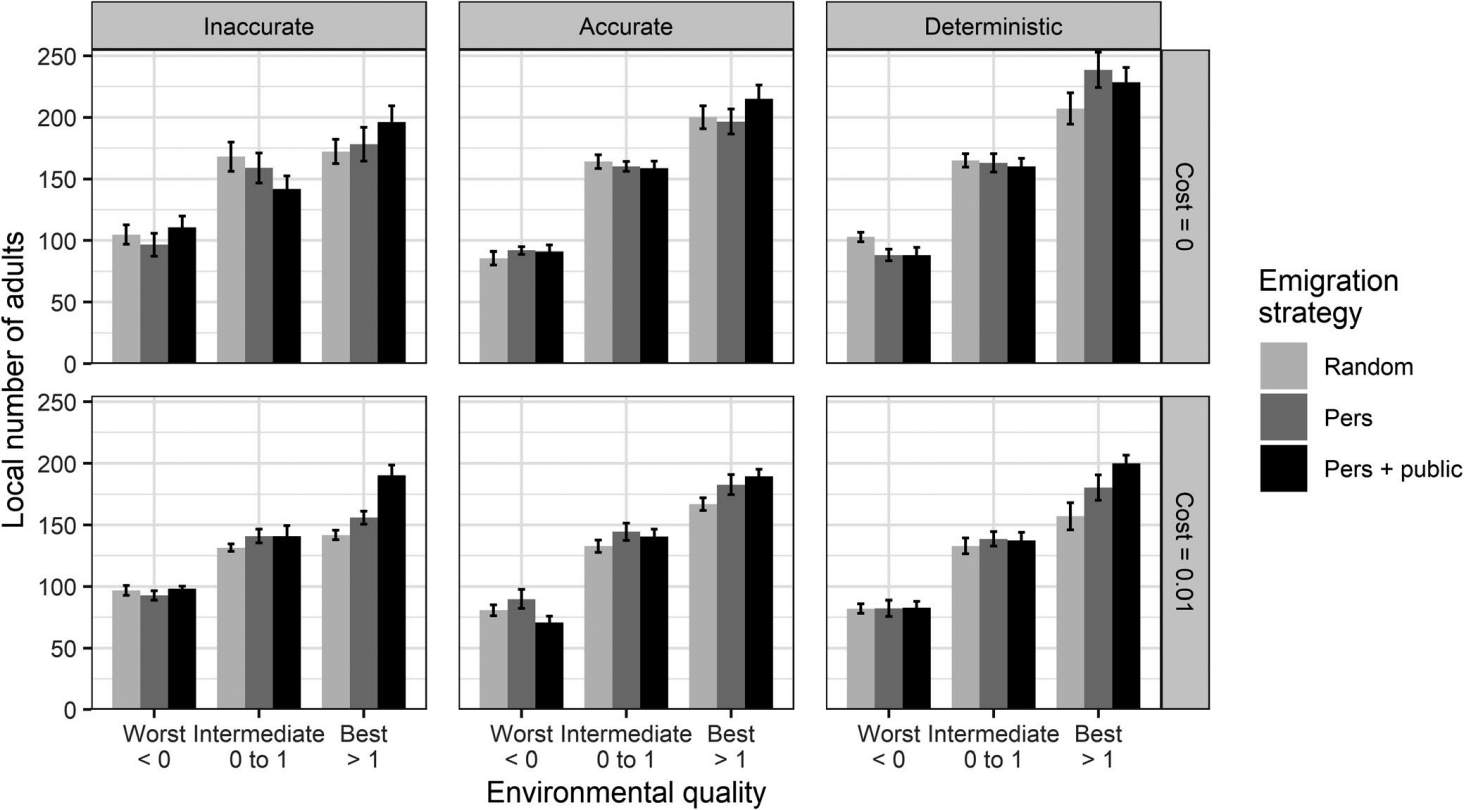
Local number of adults (±SE) depending on the local environmental quality, emigration strategy, patch selection process, and per‐patch prospecting mortality cost. Pers is the use of personal information (individual breeding performance). Pers + public is the use of both personal and public information (conspecific breeding success) in emigration decisions. Results are shown after 20,000 years over 10 replicates

Independently from the cost of prospecting, emigration strategy influences individual distribution in the patches according to their local environmental quality (Figure 2). In general, while densities are similar in patches of intermediate quality among scenarios, they are lower in bad patches and higher in good patches. The more accurate the information used to select their new breeding patch is (patch selection process), the higher the number of individuals settling in good patches.

**FIGURE 3:**
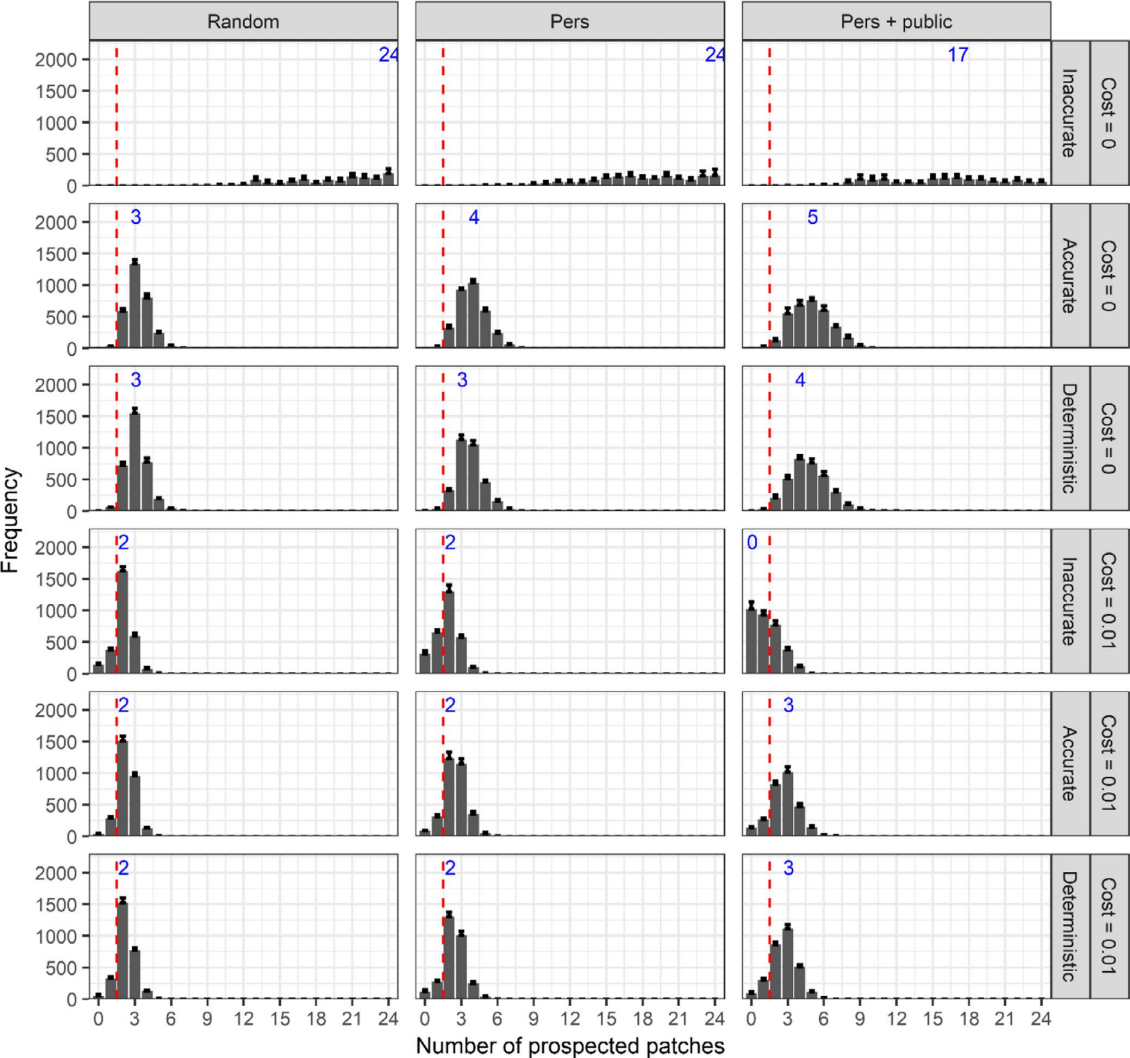
Mean (+SE) frequency of the number of prospected patches after 20,000 years over 10 replicates according to emigration strategy (columns), patch selection process, and prospecting cost per patch (rows) when only prospecting evolves. Bars left of the dashed red line correspond to random settlement (no prospecting). Blue numbers indicate the mode of the distributions

When the patch selection process is inaccurate and prospecting is costless, strategies prospecting a high number of patches evolve (Figure 3). Contrastingly, with a deterministic or accurate selection process, selection favors individuals that prospect a limited number of patches (*N*_p_ < 5). Indeed, if individuals prospected all the available patches and discriminated the best patches, they would all pile up in the same best patch. This would drastically affect their subsequent breeding success through negative density‐dependent effects on their fecundity, and there would be a dynamic imbalance in distribution of overcrowded good patches and underpopulated bad patches. When prospecting entails a mortality cost and individuals discriminate the best patches, prospecting is still favored over random dispersal, which is not the case when individuals apply an inaccurate patch selection process with personal and public information use (Figure 3). Emigration strategy has a weak effect on the evolution of prospecting, as the modal number of prospected patches increases by one when individuals use both personal and public information with an auccrate or deterministic patch selection process.

These results confirm that there is a trade‐off between the number of prospected patches and prospecting costs, as previously suggested by Bocedi et al. (2012). The prediction of a modest number of patches being prospected is also consistent with field observations in long‐lived colonial species. Although empirical data on prospecting movements are still scarce and potential associated costs are unknown (Ponchon et al., 2013), a few studies suggest that individuals do not prospect a large number of breeding areas. For instance, failed‐breeding black‐legged kittiwakes *Rissa tridactyla* prospected only a few cliffs among the tens available within a restricted area over a month of tracking (Ponchon, Iliszko, et al., 2017). The same trend was observed at larger spatial scales in black‐legged kittiwakes (Boulinier et al., 2016; Ponchon, Aulert, et al., 2017), northern gannets *Morus bassanus* (Votier et al., 2011), and black‐browed albatrosses *Thalassarche melanophris* (Campioni et al., 2017). Our results finally align with the theoretical study by Delgado et al. (2014) which shows that prospecting length (somehow equivalent to our number of prospected patches) decreases with increased mortality costs. Our model results, together with previous theory and empirical observations, suggest that prospecting is an effective means to avoid dispersing to a particularly poor patch but also allows individuals to find the best patches. This is due to the negative subsequent impacts of density dependence if too many individuals simultaneously choose to disperse to the best few patches.

### 4.2 Evolution of both emigration and prospecting strategies

**FIGURE 4:**
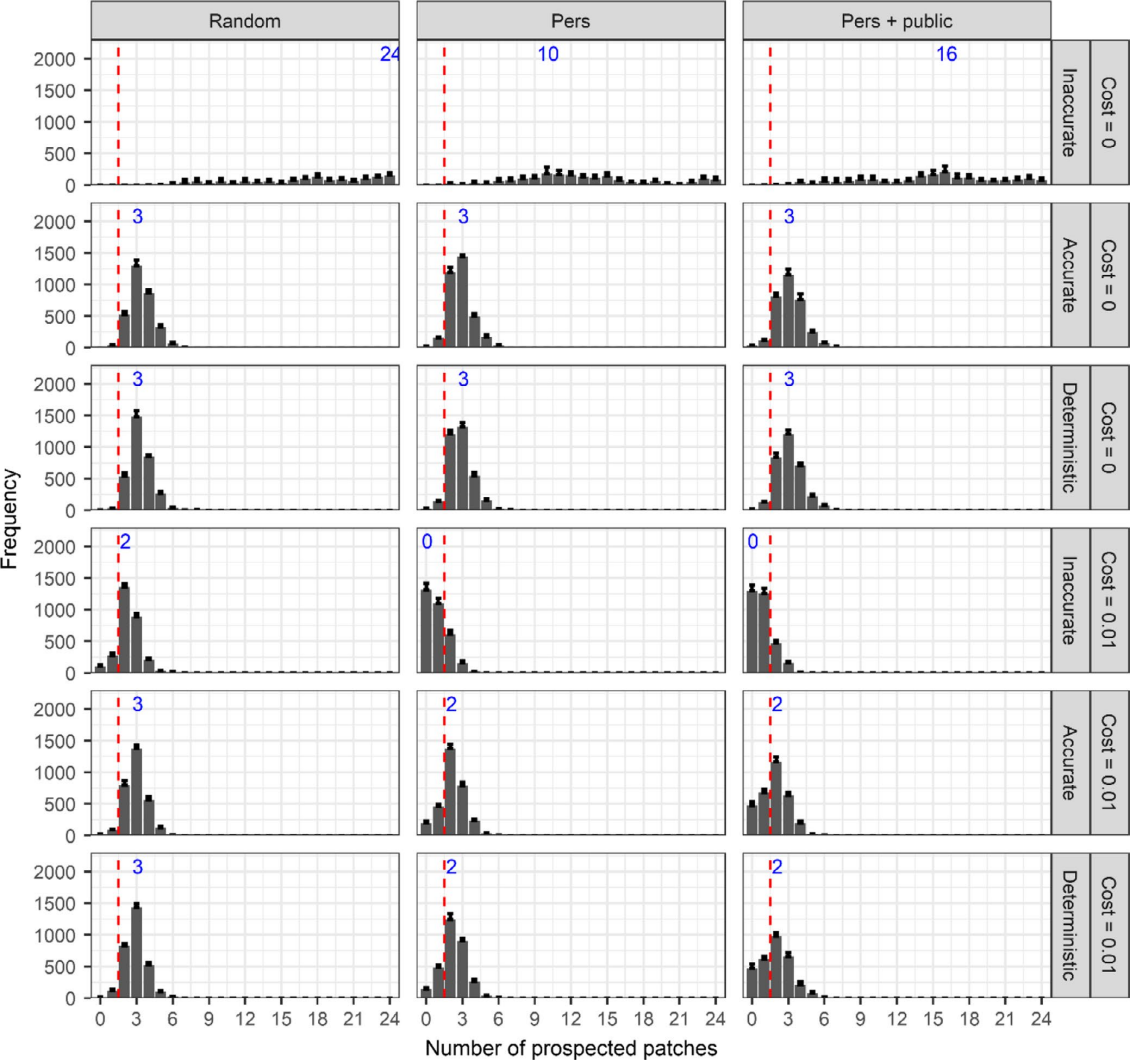
Mean (+SE) frequency of the number of prospected patches after 20,000 years over 10 replicates according to emigration strategy (columns), patch selection process, and prospecting cost per patch (rows) when both emigration and prospecting evolve. Bars left of the dashed red line correspond to random settlement (no prospecting). Blue numbers indicate the mode of the distributions

Individual distribution among patches is mostly unchanged when both prospecting and emigration probability are evolving: low densities of adults in bad patches and high densities in the best patches (Figure S5). When emigration evolves along with costless prospecting (Figure 4), individuals still evolve to prospect a low number of patches when the patch selection process is accurate or deterministic. The emigration strategy is not influential, as all strategies lead to the same number of prospecting patches (*N*_p_ = 3; Figure 3). Adding a prospecting cost slightly decreases the number of prospected patches for accurate and deterministic patch selection process, especially when individuals use information (Figure 4). With an inaccurate patch selection process and mortality cost, individuals mainly evolve to random dispersal when they use information (personal or both personal and public), but when emigration is random, prospecting based on a low number of patches is favored (Figure 4).

**FIGURE 5:**
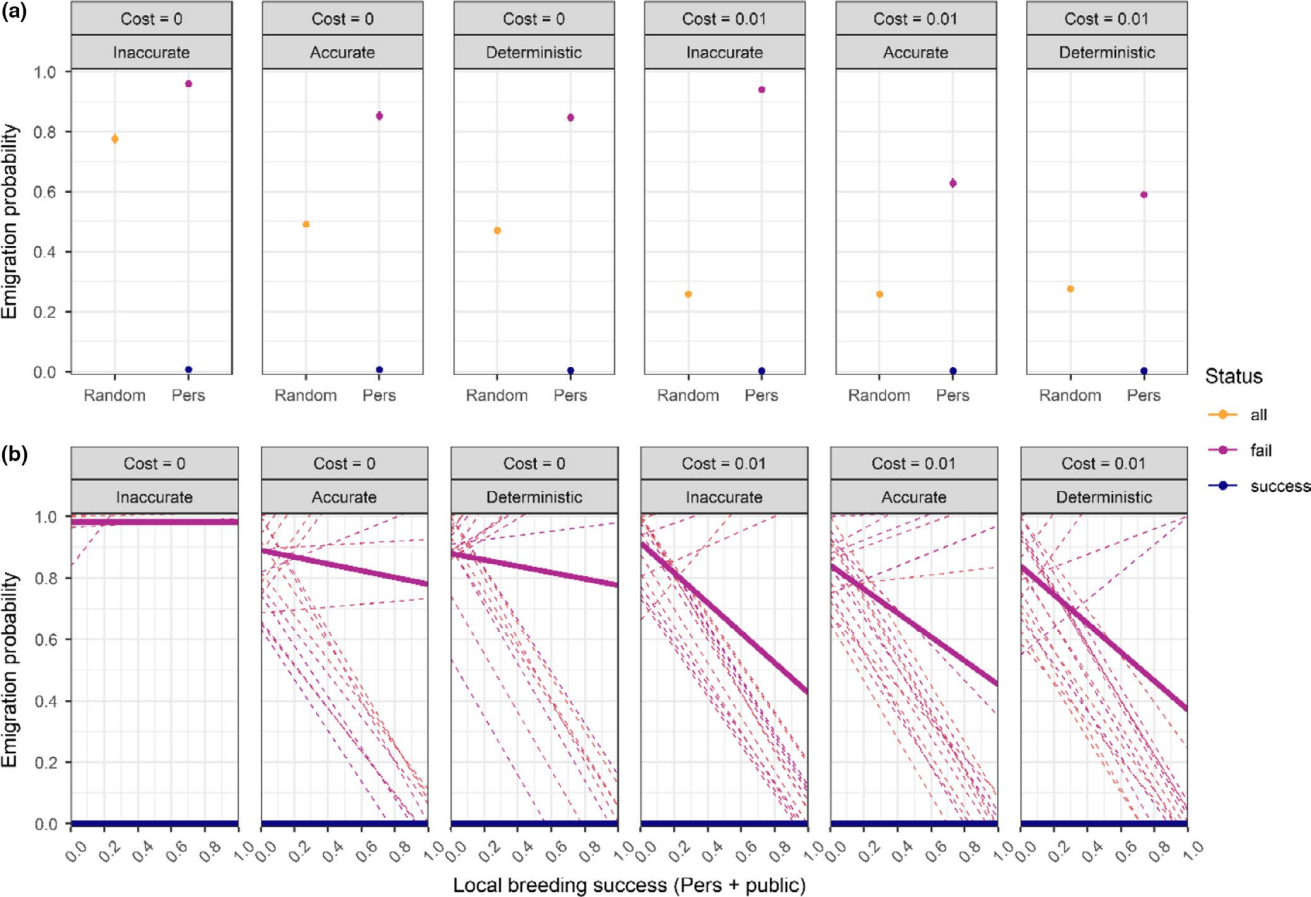
Emigration probability evolved under different patch selection processes and prospecting costs per patch after 20,000 years and over 10 replicates when both emigration probabilities and prospecting evolve. (a) Random emigration probability and emigration probability based on personal information of failed and successful breeders (error bars show ± SE) (b) Emigration probability based on personal and public information, where emigration probability is a function of the local breeding success. The bold lines represent the average reaction norm of failed breeders while thin dashed lines represent 20 random individual reaction norms. Emigration probability of successful breeders evolves to 0 independently of local breeding success

Emigration probabilities evolve very differently according to whether individuals use information in emigration decision. At the same time, accurate and deterministic patch selection strategies lead to very similar relationships between emigration probability and local breeding success (Figure 5). In the case of random emigration and no prospecting cost, emigration probability *E* evolves to 0.78 with an inaccurate patch selection strategy but decreases to 0.47–0.49 when individuals discriminate the best patches (Figure 5a). When individuals can evolve emigration probabilities conditional on breeding success, *E*_fail_ evolves to 0.95 with an inaccurate patch selection strategy and 0.85 with an accurate or deterministic strategy, while *E*_succ_ always evolves to 0 (Figure 5a), meaning that individuals do not emigrate after breeding successfully. When adding a cost to prospecting, *E* decreases to 0.26–0.28 with a random emigration, and the effect of the patch selection strategy is negligible. *E*_fail_ remains around 0.95 with an inaccurate strategy but decreases to 0.59–0.63 with an accurate or deterministic strategy. Finally, when individuals can evolve emigration probabilities conditional on both personal and public information, *E*_succ_ also always evolves to 0. On average the emigration probability of failed breeders evolves to be an inverse function of local conspecific breeding success except with an inaccurate strategy and in the absence of prospecting cost, where failed individuals tend to always disperse (Figure 5b).

Our general results align with Enfjäll and Leimar (2009), who found that the use of conspecific densities as information led to decreased emigration rate because individuals were less prone to emigrate from high habitat quality and low conspecific densities. Here, we additionally show that the use of both personal and public information for emigration probabilities is crucial, since it completely suppresses emigration when individuals breed successfully and strongly increases it when individuals fail breeding, especially when conspecific breeding success is low and when prospecting is costly (Figure 5). Consequently, not accounting for both personal and public information use may lead to important biases when estimating emigration probabilities, either in theoretical models or empirically.

Overall, our results highlight that the patch selection process is crucial in prospecting evolution, as it drives the number of prospected patches, while personal and public information use is crucial in the evolution of emigration strategies as it completely suppresses emigration when individuals breed successfully.

## 5 PERSPECTIVES

By modeling a virtual long‐lived colonial species living in a heterogeneous but temporally auto‐correlated environment, we demonstrate that informed dispersal can change the eco‐evolutionary dynamics of spatially structured populations. The modeling approach proposed here can be adapted and extended in many ways to address more specific questions on the eco‐evolutionary dynamics of informed dispersal involving prospecting.

First, some spatial complexity may be added, such as explicitly modeling individual breeding sites within patches and explicitly accounting for distances between sites and patches. This may thereby integrate the notion of perceptual range (e.g., Delgado et al., 2014). Indeed, habitat quality may vary across a hierarchy of spatial scales, and the proportion and distribution of good breeding sites available in a patch are likely to affect the spatial scale of prospecting and dispersal movements and thereby the spatial dynamics of populations (Boulinier & Danchin, 1997; Ponchon, Chambert, et al., 2015; Ponchon, Iliszko, et al., 2017). At the same time, as prospecting and subsequent dispersal movements at larger spatial scales might be risky and time‐consuming for individuals (Stamps et al., 2005), there may be important trade‐offs between the adverse environmental factors to escape (predation, lack of food, bad breeding site, etc.) and the distance covered for prospecting and subsequent dispersal movements (Baguette & Van Dyck, 2007). Hence, prospecting could even occur within one breeding patch (e.g., Ponchon, Iliszko, et al., 2017). Despite its importance in understanding individual responses to environmental variability, the hierarchical spatial aspect of the environment is often overlooked in a dispersal context and needs to be better implemented in theoretical models (Gaillard et al., 2010). Settlement decisions could also be weighted according to the timing of their use (memory effect). For instance, if an individual gathers information one year and uses it only two or more years later without updating it, there will potentially be a mismatch between the value of the information and the actual local quality of the patch. It will potentially reduce individual reproductive success, and this may have some evolutionary consequences (Bocedi et al., 2012; McNamara et al., 2011).

Here, we have implemented prospecting as a decision‐making process based on a “best‐of‐n” strategy, where individuals select the best patch from the *n* patches they have prospected (Figure 1; Path 1). It would be interesting to compare this strategy with a sequential sampling process, where individuals continue searching for a patch if the encountered one does not reach their selectivity threshold (i.e., Stamps et al., 2005; Figure 1; Path 2). Previous studies conducted in mate search and choice theory have shown that sequential sampling was more beneficial for individuals to select better mates compared to the best‐of‐n strategy, especially when accounting for search costs (Ferreira et al., 2018; Real, 1990). This might be the same with informed dispersal, but this has not yet been tested (Arendt, 2015). By modifying the implementation of the prospecting process, our model may provide an opportunity to obtain further insights into how settlement decisions made through different prospecting strategies evolve and affect population dynamics and structure.

Third, this type of model may help address the evolutionary consequences of sex‐specific prospecting and dispersal strategies. Indeed, it is still difficult to empirically relate prospecting movements and dispersal decisions at large spatial scales, and it is still not clear whether males and females display the same prospecting patterns and favor the use of the same information sources (e.g., Doligez et al., 1999; Morinay et al., 2018; Ponchon, Iliszko, et al., 2017; Schuett et al., 2012; Ward, 2005). Our approach might help explore new hypotheses in this context by implementing evolving sex‐dependent prospecting and dispersal rules. Additionally, existing theory has highlighted the importance of inbreeding in driving the evolution of sex‐biased dispersal as a means of avoiding inbreeding depression (Guillaume & Perrin, 2009; Henry et al., 2016; Perrin & Mazalov, 2000). In this context, our proposed approach could be modified to implement information on population relatedness structure as information which could be used by individuals in emigration and settlement decisions.

Fourth, the model is stage‐structured and has overlapping generations. Hence, it will be possible to compare the evolution of informed dispersal for species with different life cycles. This aspect has recently been highlighted as critical in the evolution of dispersal (Massol & Débarre, 2015). Moreover, informed natal dispersal, during which prospecting helps individuals select the patch where they will breed for the first time, might be important. As the choice of the first breeding site is likely to affect the timing of recruitment, first breeding attempt, and thus age at first reproduction (Bosman et al., 2013; Fay et al., 2016; Hadley et al., 2006), the joint evolution of prospecting and emigration is expected to affect both population structure and individual life‐history traits, especially in long‐lived species.

Fifth, physiological, morphological, or behavioral traits may influence informed dispersal. For example, several empirical studies have recently demonstrated that dispersal is likely influenced by individual personality (Cote et al., 2010; Korsten et al., 2013), and this trait has begun to be incorporated in modeling inter‐individual variation in dispersal behaviors (Fogarty et al., 2011; Palmer et al., 2014). We may expect bold individuals to explore their environment more (Martins et al., 2012), travel further (Palmer et al., 2014), and be more prone to dispersal compared to shyer individuals (Dingemanse et al., 2003). Bold individuals may thereby be more likely to display prospecting strategies (Burkhalter et al., 2015 but see Schuett et al., 2012). The evolutionary consequences of personality on individual decisions, movements, and realized dispersal have already been addressed (Burkhalter et al., 2015; Cote et al., 2010; Duckworth, 2008; Duckworth & Badyaev, 2007), but public information use has never been explicitly included. Addressing them with our modeling approach may provide new hypotheses that could be later tested in the field.

Finally, ecological models will help to predict better the spatial distribution and population dynamics of species using informed dispersal (e.g., Ponchon, Garnier, et al., 2015). They will also help refine conservation strategies based on the use of decoys and playbacks to attract individuals to targeted restored or newly suitable breeding areas (e.g., Ahlering et al., 2010; James et al., 2015; VanderWerf et al., 2019).

## 6 CONCLUSION

The modeling approach proposed here offers new avenues both for theoretical and for applied purposes, as it can be developed for species displaying various life‐history traits and informed dispersal strategies. Building further theory and generating predictions from this new modeling approach can provide a powerful means to understand the potential range of individual and population responses to environmental change within a context of informed dispersal and may ultimately improve our capability for predicting species responses to environmental changes (Cote et al., 2017; Kokko & Lopéz‐Sepulcre, 2006; Ponchon, Garnier, et al., 2015; Urban et al., 2016).

## CONFLICTS OF INTEREST

The authors declare no conflict of interest.

## AUTHOR CONTRIBUTIONS

**Aurore Ponchon:** Conceptualization (lead); formal analysis (lead); funding acquisition (lead); investigation (lead); methodology (lead); project administration (lead); validation (lead); visualization (lead); writing—original draft (lead); writing—review and editing (lead). **Alice Scarpa:** formal analysis (supporting); investigation (supporting); methodology (supporting); validation (supporting); writing—review and editing (supporting). **Greta Bocedi:** methodology (supporting); validation (supporting); writing—review and editing (supporting). **Steve C. F. Palmer:** methodology (supporting); validation (supporting); writing—review and editing (supporting). **Justin M. J. Travis:** conceptualization (supporting); funding acquisition (supporting); methodology (supporting); supervision (supporting); validation (supporting); writing—review and editing (supporting).

### OPEN RESEARCH BADGES

This article has been awarded Open Data, Open Materials Badges. All materials and data are publicly accessible via the Open Science Framework at https://github.com/auponchon/Informed‐dispersal‐IBM; https://doi.org/10.5281/zenodo.5534084.

## Supporting information

Figures S1–S5

## Data Availability

The source code and output files are freely accessible on the GitHub repository https://github.com/auponchon/Informed‐dispersal‐IBM and are deposited on Zenodo (https://doi.org/10.5281/zenodo.5534084).
