# Supplementary material for: Prospecting and informed dispersal: Understanding and predicting their joint eco‐evolutionary dynamics
Source: Ecol Evol. 2021 Oct 18;11(21):15289–302. doi: 10.1002/ece3.8215 (PMC8571608; doi:10.1002/ece3.8215)

**Prospecting and informed dispersal: understanding and predicting their joint eco-evolutionary dynamics.**

Aurore Ponchon, Alice Scarpa, Greta Bocedi, Stephen CF Palmer, Justin MJ Travis

School of Biological Sciences, University of Aberdeen, Zoology Building, Tillydrone Avenue, Aberdeen, AB24 2TZ, UK

**Supplemental material**

**Figure S1:** Example of the variability of the environmental quality of 4 patches over time according to different auto-correlation coefficients α (0.1 is a low temporal auto-correlation; 0.9 is a high temporal auto-correlation) and different standard deviation σ.

1. Standard deviation σ=0.5


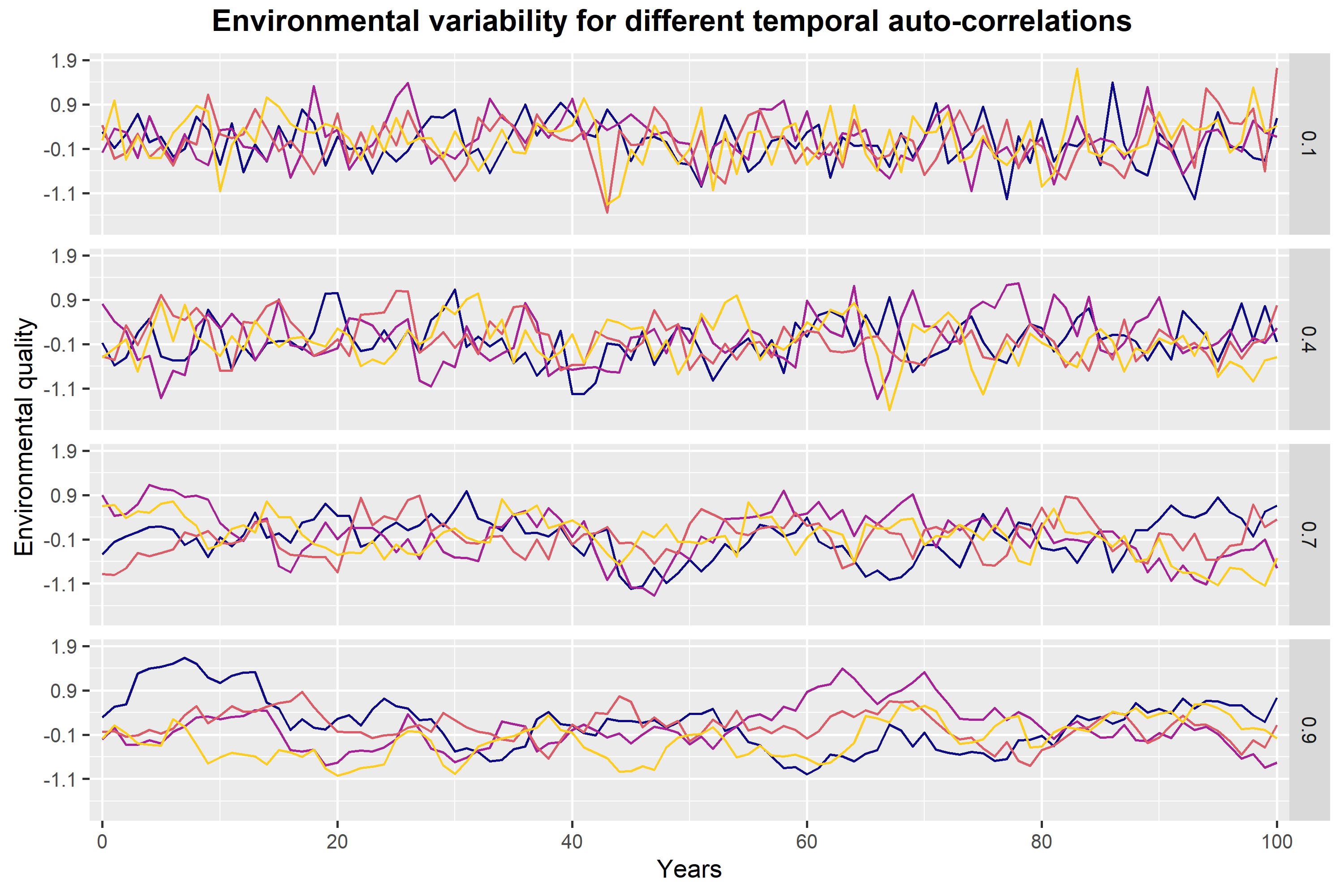


1. Standard deviation σ=1


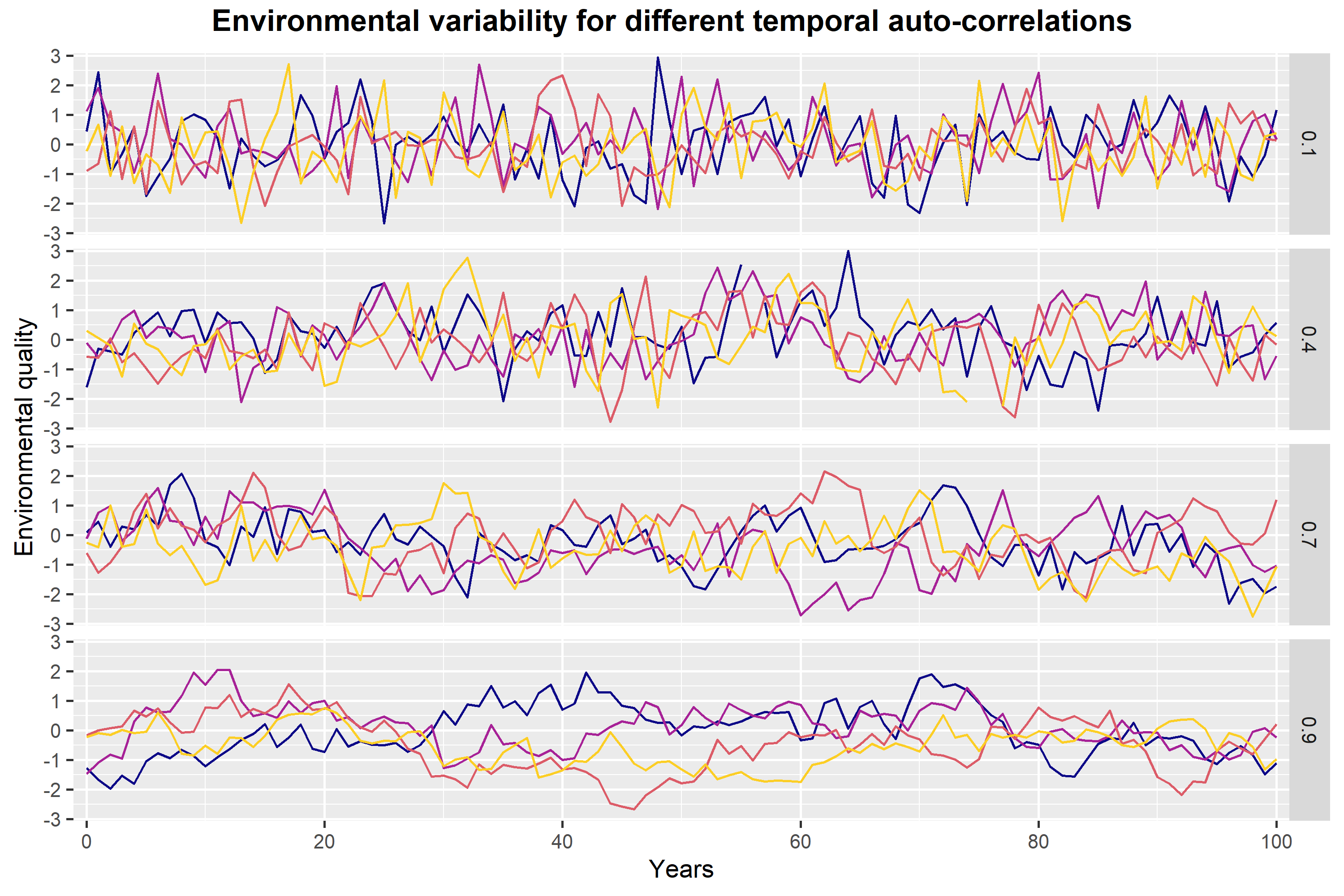


1. Standard deviation σ=2


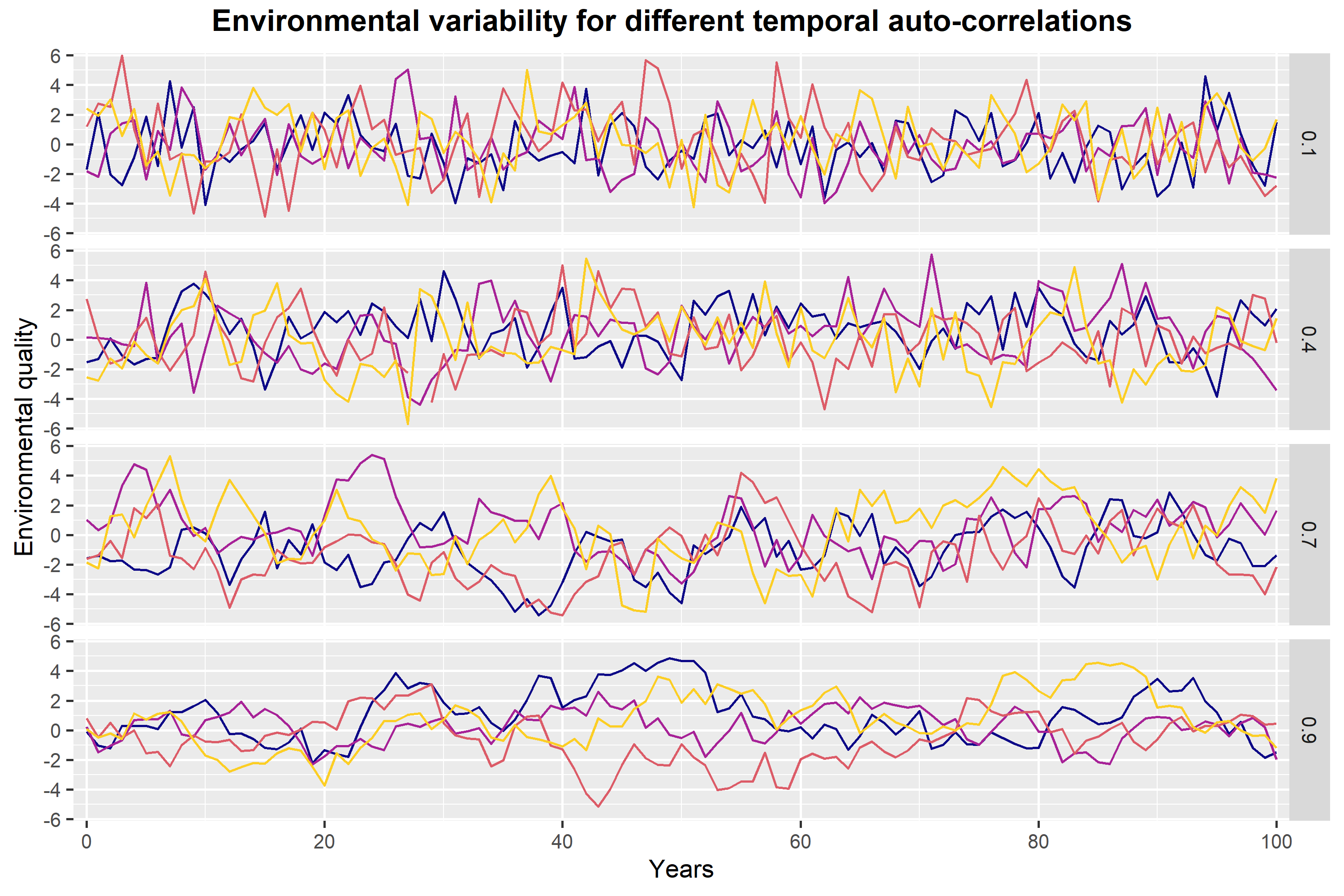


**Figure S2:** Expected number of offspring according to emigration strategy, patch selection process and auto-correlation coefficient when only prospecting evolves in an environment. Each point represent one patch.

1. Standard deviation σ=0.5


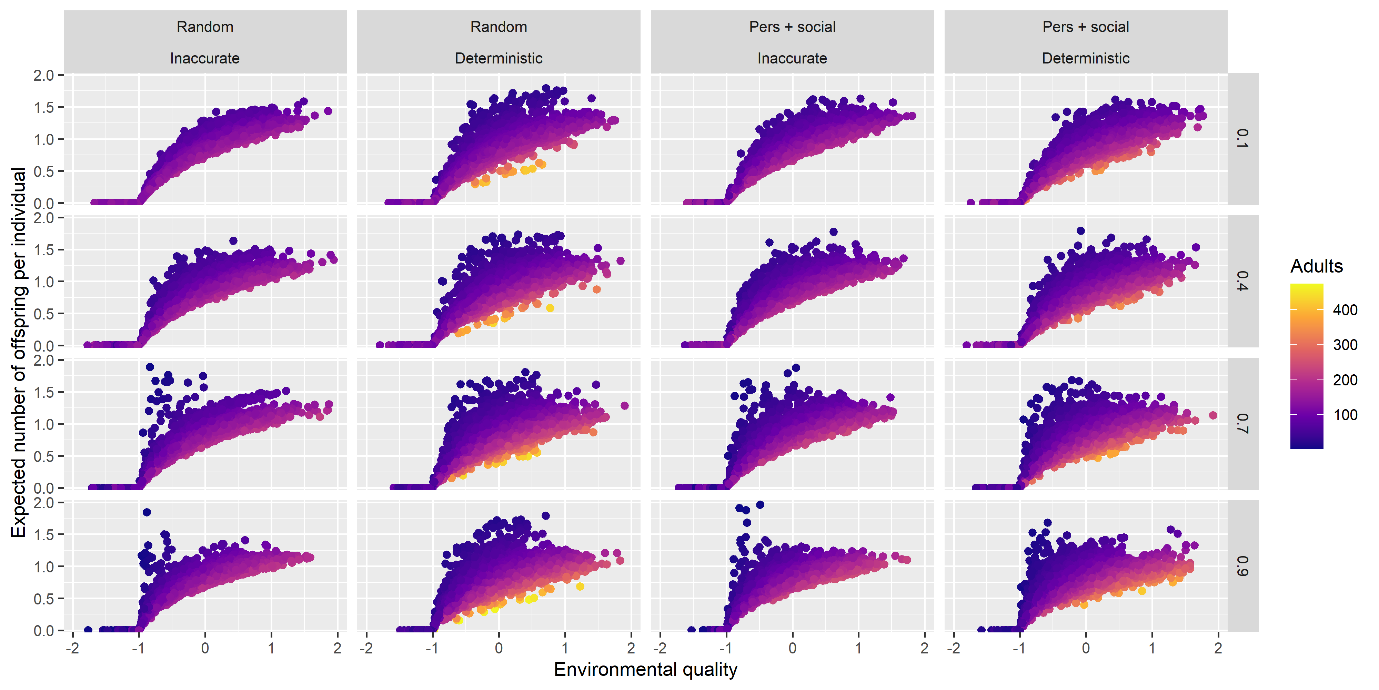


1. Standard deviation σ=1


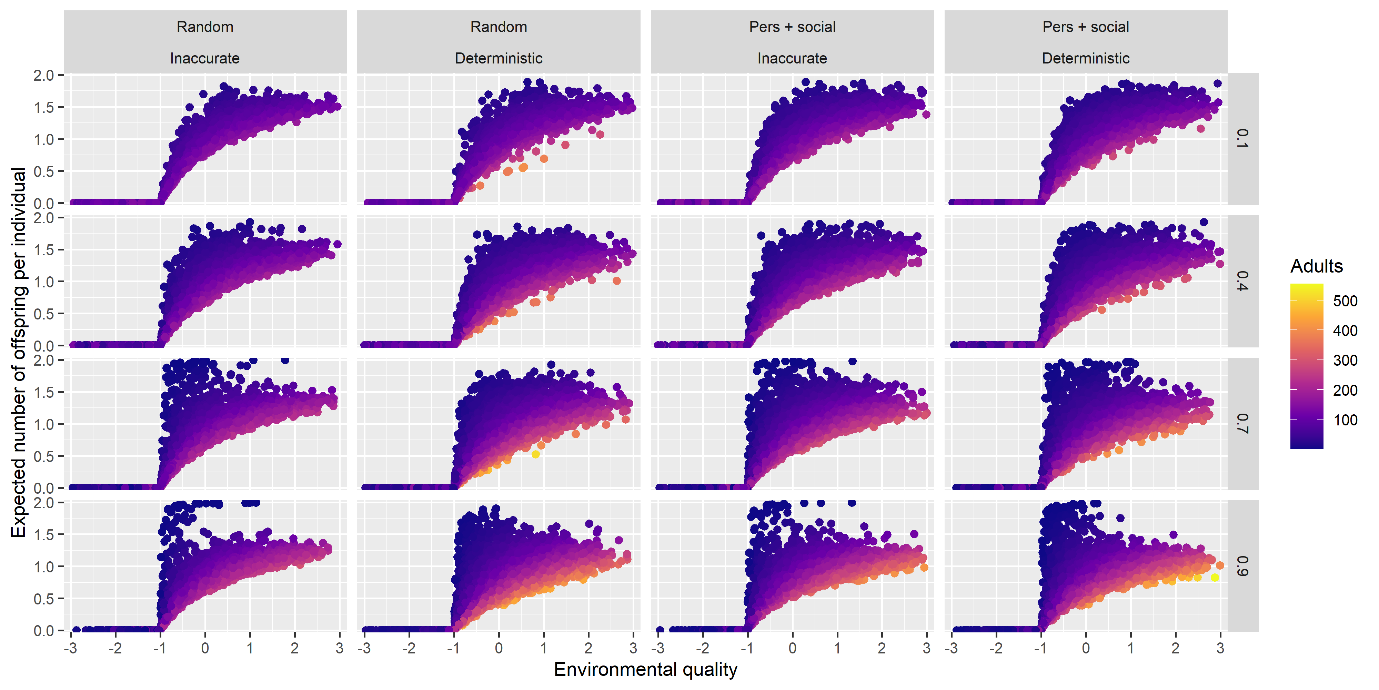


1. Standard deviation σ=2


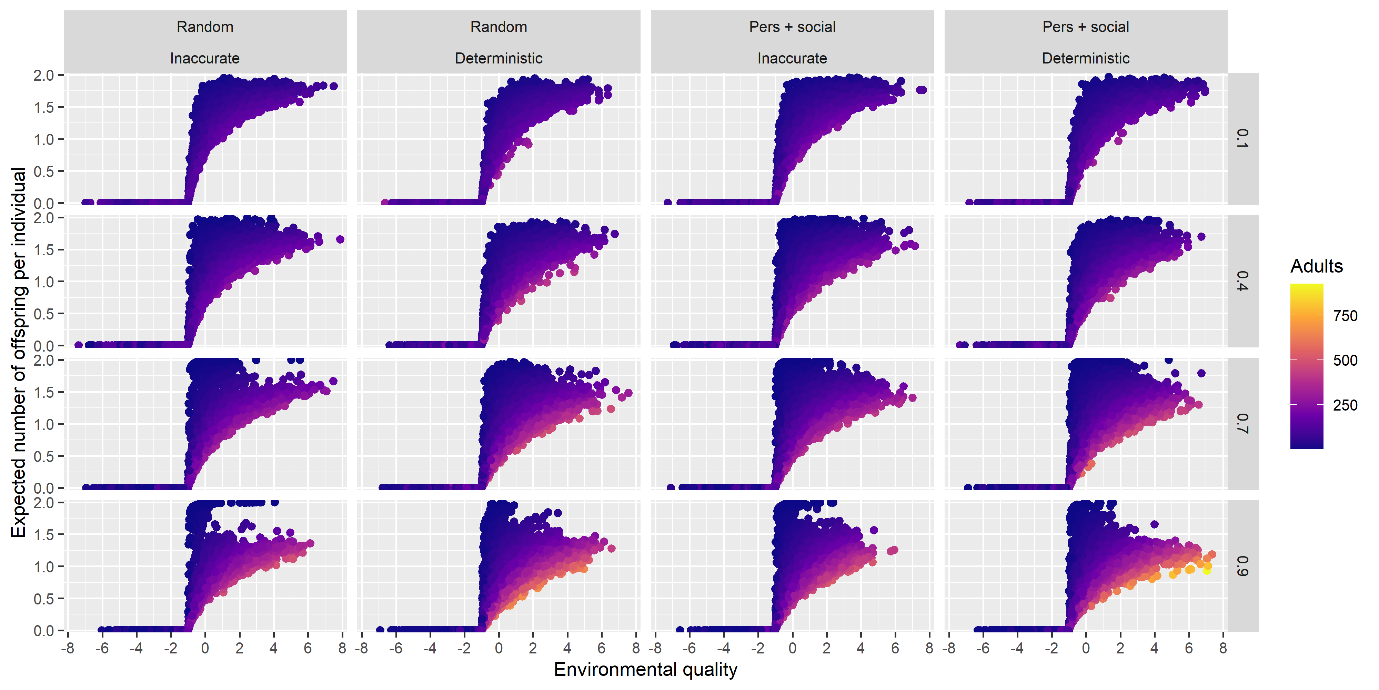


**Figure S3:** Mean (+ SE) frequency of the number of prospected patches after 20000 years over 10 replicates according to emigration strategy, patch selection process and auto-correlation coefficient when only prospecting evolves in an environment which standard deviation σ=0.5. Bars left of the dashed red line correspond to random settlement (no prospecting). Blue numbers indicate the mode of the distributions.

1. Standard deviation σ=0.5


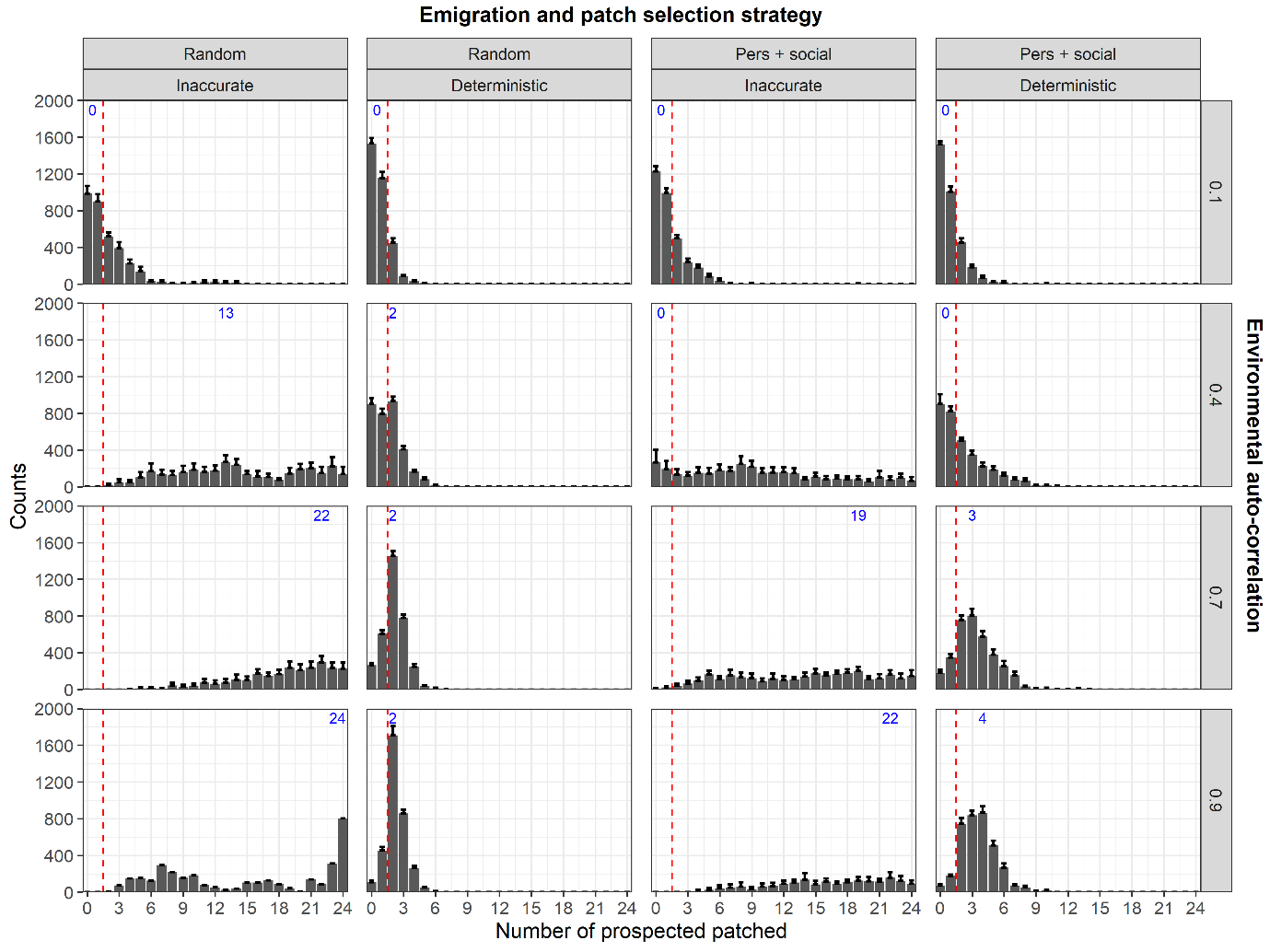


1. Standard deviation σ=1


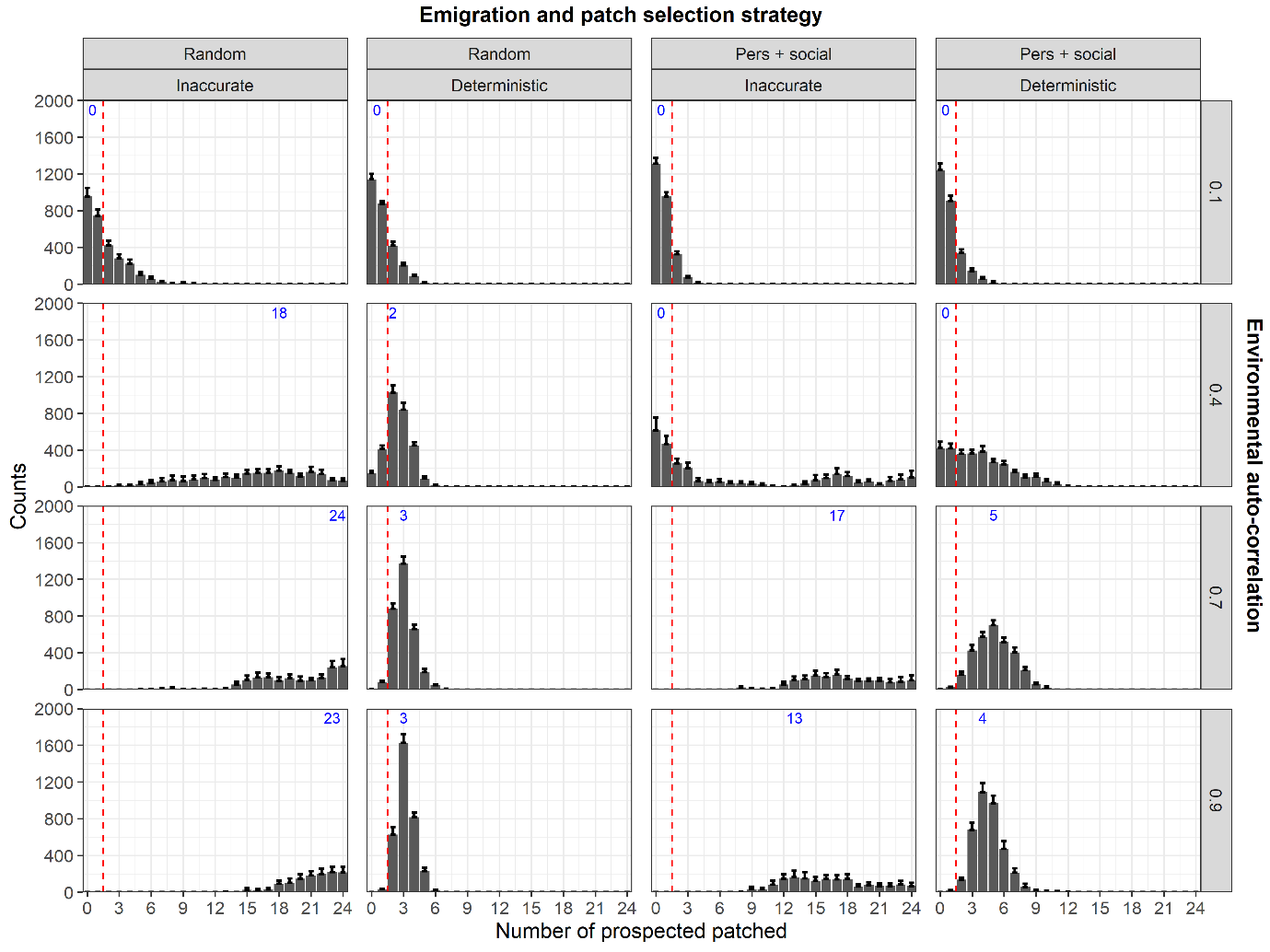


1. Standard deviation σ=2


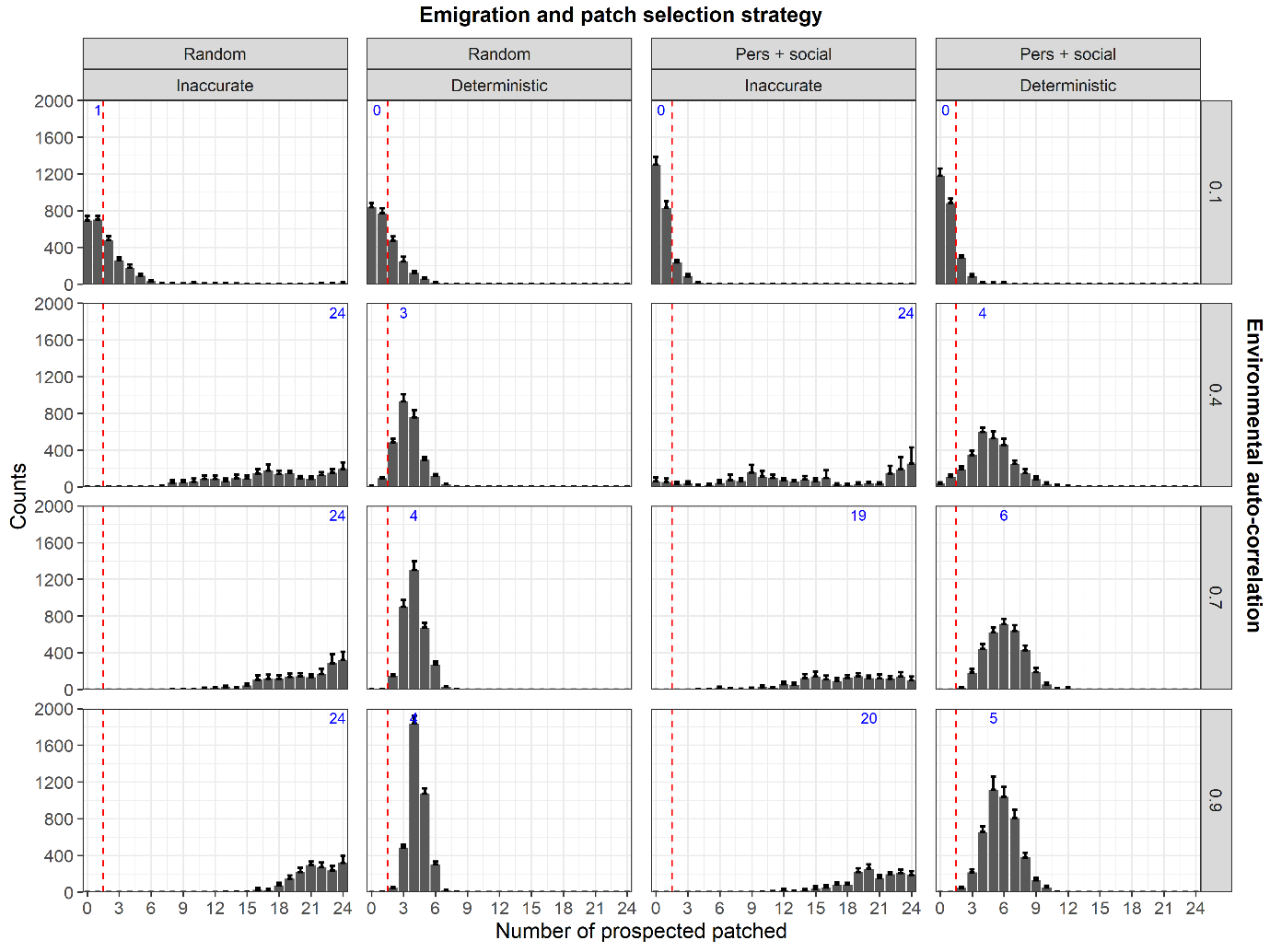


**Figure S4:** Cumulative probabilities for dispersing individuals to choose a prospected patch. Patches are ranked based on their local breeding success. Note that in the deterministic patch selection process, all individuals always select the first patch, which has the highest local breeding success.


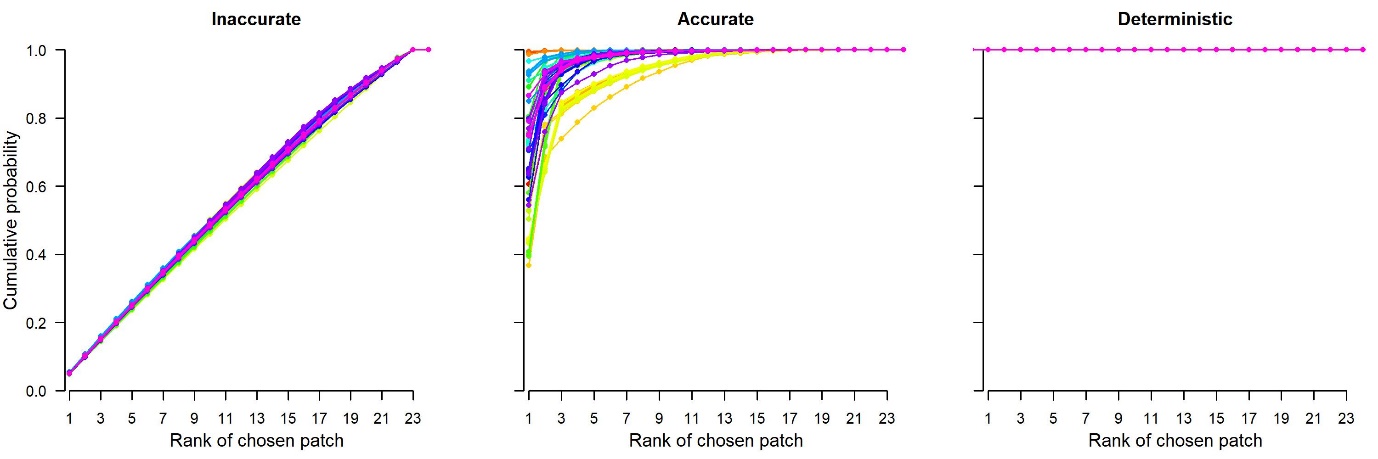


**Figure S5:** Local number of adults (± SE) depending on the local environmental quality, emigration strategy, patch selection process and prospecting cost when both prospecting and emigration probability evolve. Pers is the use of personal information (individual breeding performance). Pers+public is the use of both personal and public information (conspecific breeding success) in emigration decisions. Results are shown after 20000 years over 10 replicates.


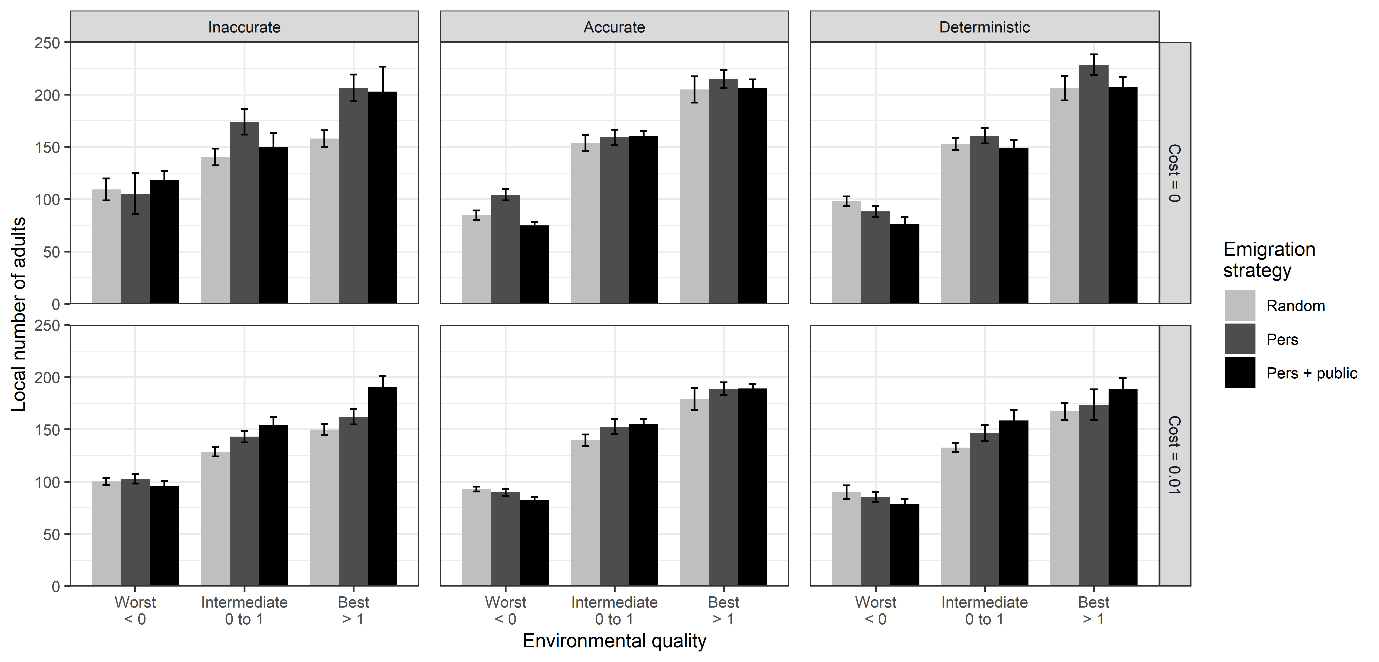

Supplement: Supplementary file 1 — Figures S1–S5 [file ECE3-11-15289-s001.docx]
